# Demographic, clinical, and treatment characteristics of the juvenile primary fibromyalgia syndrome cohort enrolled in the Childhood Arthritis and Rheumatology Research Alliance Legacy Registry

**DOI:** 10.1186/s12969-019-0356-z

**Published:** 2019-07-26

**Authors:** Jenifer E. Weiss, Kenneth N. Schikler, Alexis D. Boneparth, Mark Connelly, For the CARRA Registry Investigators, L. Abramson, E. Anderson, M. Andrew, N. Battle, M. Becker, H. Benham, T. Beukelman, J. Birmingham, P. Blier, A. Brown, H. Brunner, A. Cabrera, D. Canter, D. Carlton, B. Caruso, L. Ceracchio, E. Chalom, J. Chang, P. Charpentier, K. Clark, J. Dean, F. Dedeoglu, B. Feldman, P. Ferguson, M. Fox, K. Francis, M. Gervasini, D. Goldsmith, G. Gorton, B. Gottlieb, T. Graham, T. Griffin, H. Grosbein, S. Guppy, H. Haftel, D. Helfrich, G. Higgins, A. Hillard, J. R. Hollister, J. Hsu, A. Hudgins, C. Hung, A. Huttenlocher, N. Ilowite, A. Imlay, L. Imundo, C. J. Inman, J. Jaqith, R. Jerath, L. Jung, P. Kahn, A. Kapedani, D. Kingsbury, K. Klein, M. Klein-Gitelman, A. Kunkel, S. Lapidus, S. Layburn, T. Lehman, C. Lindsley, M. MacgregorHannah, M. Malloy, C. Mawhorter, D. McCurdy, K. Mims, N. Moorthy, D. Morus, E. Muscal, M. Natter, J. Olson, K. O’Neil, K. Onel, M. Orlando, J. Palmquist, M. Phillips, L. Ponder, S. Prahalad, M. Punaro, D. Puplava, S. Quinn, A. Quintero, C. Rabinovich, A. Reed, C. Reed, S. Ringold, M. Riordan, S. Roberson, A. Robinson, J. Rossette, D. Rothman, D. Russo, N. Ruth, K. Schikler, A. Sestak, B. Shaham, Y. Sherman, M. Simmons, N. Singer, S. Spalding, H. Stapp, R. Syed, E. Thomas, K. Torok, D. Trejo, J. Tress, W. Upton, R. Vehe, E. von Scheven, L. Walters, J. Weiss, P. Weiss, N. Welnick, A. White, J. Woo, J. Wootton, A. Yalcindag, C. Zapp, L. Zemel, A. Zhu

**Affiliations:** 1 Hackensack University Medical Center, 30 Prospect Ave, Hackensack, NJ 07601 USA; 2 University of Louisville School of Medicine, Louisville, KY 40292 USA; 3 New York-Presbyterian Medical Center, New York, NY 10032 USA; 4 Children’s Mercy Kansas City, 2401 Gillham Road, Kansas City, MO 64108 USA

**Keywords:** Juvenile fibromyalgia, Pain, Adolescent, CARRA, Registry

## Abstract

**Background:**

To describe the demographic, clinical, and treatment characteristics of youth diagnosed with juvenile primary fibromyalgia syndrome (JPFS) who are seen in pediatric rheumatology clinics.

**Methods:**

Information on demographics, symptoms, functioning, and treatments recommended and tried were obtained on patients with JPFS as part of a multi-site patient registry (the Childhood Arthritis and Rheumatology Research Alliance Legacy Registry). Data were summarized using descriptive statistics. In a subset of patients completing registry follow-up visits, changes in symptoms, pain, and functioning were evaluated using growth modeling.

**Results:**

Of the 201 patients with JPFS enrolled in the registry, most were Caucasian/White (85%), non-Hispanic (83%), and female (84%). Ages ranged from 9 to 20 years (M = 15.4 + 2.2). The most common symptoms reported were widespread musculoskeletal pain (91%), fatigue (84%), disordered sleep (82%), and headaches (68%). Pain intensity was rated as moderate to severe (M = 6.3 + 2.4/10). Scores on measures of functioning indicated mild to moderate impairment, with males observed to report significantly greater impairments. For the 37% of the initial cohort having follow-up data available, indicators of function and well-being were found to either worsen over time or remain relatively unchanged.

**Conclusions:**

The symptoms of JPFS remained persistent and disabling for many patients treated by pediatric rheumatologists. Further study appears warranted to elucidate gender differences in the impact of JPFS symptoms. Work also is needed to identify accessible and effective outpatient treatment options for JPFS that can be routinely recommended or implemented by pediatric rheumatology providers.

## Background

Juvenile primary fibromyalgia syndrome (JPFS) is an idiopathic chronic pain syndrome thought to affect up to 6% of children and adolescents [1–4]. Clinical features of the condition were first described by Yunus and Masi in 1985 based on a sample of 33 children [5]. JPFS was characterized at that time as a pediatric condition primarily comprising widespread musculoskeletal pain, tender points at soft tissue sites, fatigue, poor sleep, headaches, paresthesia, and anxiety [5]. Contemporary understanding of how JPFS manifests has continued to be derived primarily from small samples of almost exclusively female patients seen in a few practices [2–4]. Criteria for what should constitute a diagnosis of JPFS also have been debated and variably applied since the original Yunus and Masi report [2, 6]. Conclusions from past studies on the impact and outcomes of JPFS therefore may not be applicable to the entire spectrum of this heterogeneous population.

Knowledge about effective treatments for JPFS also remains underdeveloped. As a result, wide practice variations may exist in how pediatric rheumatologists treat patients with JPFS. A variety of classes of medications (e.g., opioids, non-opioid analgesics, anticonvulsants, antidepressants, muscle relaxants) have been tried for treating symptoms of JPFS despite limited efficacy studies [7]. Nonpharmacological treatments, particularly physical therapies (PT) and training in cognitive-behavioral pain coping skills, have more established empirical support for improving symptoms and functioning in JPFS [8, 9]. However, these approaches may not be routinely recommended by pediatric rheumatologists due to limited awareness and/or availability.

Improving knowledge about the full spectrum of characteristics and approaches to treatment of JPFS requires patient data from multiple clinical sites. The Childhood Arthritis and Rheumatology Research Alliance (CARRA) Legacy Registry is a prospective observational registry comprising over 9000 youth with rheumatic disease across 50 clinical sites. The current study used data from the CARRA Legacy Registry to: (a) establish the demographic and symptom characteristics of youth with JPFS presenting to pediatric rheumatologists; and (b) identify commonalities and variances in treatment recommendations for JPFS initiated by pediatric rheumatologists. In a subset of patients who returned for follow-up visits, we also sought to determine the extent to which symptoms and functioning change over time.

## Methods

### Study design

We conducted a retrospective review of data from the CARRA Legacy Registry of JPFS patients enrolled between 2010 and 2014. Eligible patients fulfilled either the 1990 American College of Rheumatology (ACR) [10] or Yunus and Masi [5] diagnostic criteria for JPFS (depending on age at time of enrollment), were < 18 years at the time of symptom onset, and were < 21 years at the time of enrollment. Study questionnaires were in English. Children were eligible for enrollment at any time during their disease course. Written informed consent and assent (for patients > 9 years old) were obtained for all subjects.

A general and condition-specific standardized case report form (CRF) was used across registry sites to uniformly collect data on the study measures. Recorded information was manually entered into a registry database by site research coordinators. These data then were securely pooled and stored in an i2b2 data warehouse accessible by approved registry site investigators [11]. The current study was approved by the Institutional Review Board at Hackensack University Medical Center.

### Measures

#### Baseline visit measures

##### Patient characteristics

Information on self- or parent-reported demographics (ethnicity, race, sex, and family household income), diagnosis, date of symptom onset, and date first seen by a pediatric rheumatologist was recorded on standardized CRF at the baseline visit. Patient anthropometrics were recorded and converted to Body Mass Index (BMI).

##### Symptoms

Clinicians completed checklists intended to determine the extent to which common symptoms of JPFS were present in the month leading up to the baseline visit. Checklist items included widespread musculoskeletal pain (bilateral pain locations above and below the waist), frequent headaches, irritable bowel symptoms, subjective soft tissue swelling of the extremities, numbness and tingling of the extremities, pain modulation with physical activity, pain modulation with weather changes, pain modulation by anxiety or stress, a comorbid anxiety and/or depressive disorder, and disordered sleep. If disordered sleep was endorsed, patients were asked to further specify whether they had non-restorative sleep, increased sleep latency, hypersomnia, and/or frequent waking. The presence of hypermobility was recorded if the patient had a Beighton score > 4 on physical exam [12]. The Beighton score is a valid instrument to evaluate generalized joint mobility in school–aged children [13].

##### Past treatments

Information was documented on medications and non-pharmacologic treatments the patient had tried prior to the initial registry visit. Medications were categorized as nonsteroidal anti-inflammatory drugs (NSAIDs), selective serotonin reuptake inhibitors (SSRIs), tricyclic antidepressants, serotonin norepinephrine reuptake inhibitors (SNRIs), gamma-aminobutyric acid (GABA) analogues, and opioids. Non-pharmacologic treatments queried included PT, herbals/supplements, therapeutic massage, meditation, chiropractic, acupuncture/acupressure, yoga, hypnosis, and craniosacral therapy.

##### Pain intensity

Average pain intensity over the past week was rated by patients who were at least 10 years of age using a 0–10 numeric rating scale (NRS) (0 = “no pain” and 10 = “very severe pain”). The NRS is a well-established measure for pediatric populations [14]. Parent proxy reports (using a 0–10 NRS) were obtained in children under 10 years of age.

##### Functional ability and health-related quality of life

The Childhood Health Assessment Questionnaire (CHAQ) was completed by patients (or caregiver proxies if patients were less than 10 years of age) as a measure of the impact of fibromyalgia on daily functioning [15]. This questionnaire includes questions about functional ability over the past week across eight categories: dressing, grooming, arising, eating, walking, hygiene, reach, and grip. Item scores range from 0 to 3 and are averaged to form a total “Disability Index” score (ranging from 0 to 3), with higher scores indicating greater functional disability.

Patients (10 years or older) or caregivers also completed two global ratings pertaining to the patient’s health status [16]. On the first, patients/caregivers were asked to consider the ways their/their child’s condition affects them and rate on a 0–10 scale (“very well” to “very poor”) how the patient is doing, with higher numbers indicating poorer well-being. On the other, patients/caregivers were asked to rate the patient’s overall health-related quality of life (HRQOL) on a 5-point scale (“excellent” to “very poor”), with higher scores indicating poorer health-related quality of life. In addition, the patients’ functional ability was assessed using the ACR functional class rating. This measure is a physician rating of the patient’s functioning on a 1–4 scale ranging from “able to perform usual activities of daily living” to “limited in ability to perform usual self-care, vocational, and avocational activities.” [17]

##### Recommended treatments

Treatments recommended or provided at the baseline visit by a pediatric rheumatologist were documented by the clinician/research nurse on a CRF. Choices appearing on the form included the following: psychoeducation about chronic pain, sleep hygiene education, graduated aerobic exercise, medications for pain, PT, general counseling, cognitive-behavioral therapy (CBT), biofeedback-assisted relaxation training, and other specialty referral (including psychiatry, pain clinic, rehabilitation clinic, or integrative medicine clinic).

#### Follow-up measures

For patients completing the baseline visit and returning for a follow-up visit, the checklists of disease symptoms, pain intensity rating, and functioning and quality of life measures were repeated to gauge stability of these variables over time. Patient adherence to treatment recommendations was also documented, along with reasons for the patient/family not pursuing recommendations if applicable (i.e., service not available, not covered by insurance, patient/family disinterest, or “other”).

### Statistical analyses

Descriptive statistics (frequency counts and estimates of central tendency and variability as applicable) were used to summarize data from the baseline visit on patient demographics, symptoms, pain intensity, indices of functioning and well-being, and treatments tried and recommended. Age and sex differences in symptoms, treatments, and the indices of functioning and well-being were explored using point-biserial correlations (*r*_bs_), chi-square analyses (χ^2^), or independent samples *t*-tests as applicable. SPSS® software (IBM) version 24.0 was used for these analyses.

To evaluate changes in symptoms and functioning following the baseline visit, multilevel growth modeling using HLM version 7.0 (Scientific Software International Inc.) was used. Only those in the sample with at least one follow-up visit were included in these analyses. In these models, time elapse since the baseline visit was specified as a predictor of changes in endorsed symptoms, pain intensity, and scores/ratings on the functioning and well-being indices. For the dependent variables that were coded as binary (i.e., presence of a given symptom), a logistic model was specified with a Poisson distribution and logit link function [18]. Significant positive values for the estimated time coefficient from these models indicate a reliable increase in the likelihood of the item being endorsed (for symptoms) or increased impairment in the functioning and well-being indices; significant negative values for the time coefficient indicate the opposite. Age, sex, symptom duration, and self-reported adherence to initial recommendations were evaluated as moderators of changes over time in these variables. We found no significant concerns emerged regarding model misfit based on the model specifications we used.

## Results

### Patient and condition characteristics

Table 1 provides summary information on patient characteristics and symptoms reported at the baseline visit. Data were available from 201 patients (33 males) with JPFS enrolled across 23 unique clinical sites. Ages at the baseline visit ranged from 9 to 20 years (mean = 15.4, SD = 2.2). Most patients identified as Caucasian/White (85%) and non-Hispanic (83%). Reported annual household income was fairly evenly distributed across categories (median = $75-100 K/year). All patients except one reported having health insurance. Patient BMIs recorded at the baseline visit ranged from 13.6 to 50.6 (mean = 24.2, SD = 6.1). Patients reported being symptomatic for a mean of 1.7 years (SD = 2.2 years; range 0–11.8 years) prior to their first visit to a pediatric rheumatologist. Thirty-six patients (18%) endorsed having a family history of fibromyalgia. Greater household income was associated with less time elapse between reported initial symptom onset and evaluation with a pediatric rheumatologist (*r* = −.21, *p* = .01); no other demographic variables were significantly associated with this time elapse.

**Table 1:** Baseline demographic and clinical characteristics of patients with juvenile primary fibromyalgia syndrome in the CARRA Legacy Registry

Variable	Value
Age in years at diagnosis (Mean ± SD)	15.4 ± 2.2
Sex (*n,* %)	
Male	33 (16%)
Female	168 (84%)
Race (*n,* %)	
White	171 (85%)
Black/African-American	15 (8%)
American Indian or Alaskan Native	1 (1%)
Asian	4 (2%)
Mixed race	5 (2%)
Other	5 (2%)
Ethnicity (*n,* %)	
Not Hispanic	167 (83%)
Hispanic	34 (17%)
Annual household income (*n,* valid %)	
< $25,000	23 (15%)
$25–$49,999	18 (12%)
$50–$74,999	29 (19%)
$75–$99,999	22 (14%)
$100–$150,000	34 (22%)
> $150,000	30 (19%)
Unknown/missing	45
Body Mass Index (Mean ± SD)	24.2 ± 6.1
Symptom duration in years prior to diagnosis (Mean ± SD)	1.7 ± 2.1
Symptoms endorsed for past month	
Widespread musculoskeletal pain	164 (91%)
Pain modulation with anxiety or stress	121 (80%)
Pain modulation with physical activity	117 (75%)
Frequent headaches	111 (68%)
Pain modulation with weather change	86 (61%)
Nonrestorative sleep	94 (52%)
Frequent awakenings	75 (42%)
Increased sleep latency	74 (41%)
Numbness and tingling of extremities	48 (32%)
Anxiety and/or depression	40 (28%)
Hypermobility on exam	35 (28%)
Subjective soft tissue swelling of extremities	32 (22%)
Irritable bowel symptoms	24 (16%)
Hypersomnia	25 (14%)

The most commonly reported symptoms endorsed by patients at the baseline visit included widespread musculoskeletal pain (91%), fatigue (84%), disordered sleep (82%), and headaches (68%). Most patients also reported pain modulation by stress (80%), physical activity (75%), and weather changes (61%). There were no significant age or sex differences in symptoms endorsed except for numbness/tingling, which was more often endorsed in females than males (36% versus 13% respectively, χ^2^ = 5.09, *p* = 0.03).

Sixty-six patients (37% of the initial sample of the JPFS cohort in the registry) returned for a follow-up visit with their pediatric rheumatologist between 0.2 to 2.5 years from the baseline visit (mean = 0.9, SD = 0.5 years). The number of follow-up visits available during the time period sampled ranged from 1 (68% of the follow-up sample) to 5 (2% of the follow-up sample). Of the follow-up sample, 82% of patients were female and ranged in age from 9 to 21 years (mean = 15.0, SD = 2.28 years). There were no significant differences in known demographic characteristics between those in the initial cohort who did and did not return for follow-up visits.

### Previous treatments

Table 2 lists treatments tried as reported at the baseline visit. For pharmacological treatments, 39% of the sample reported no prior use at all, 38% reported prior use of one medication type, and 23% reported trials of at least two medication types. The most common medication previously tried was NSAIDs (27%). The least common medication tried was opioids (4%). There was no relationship of age with prior use of any of the medication categories. The only sex difference was that a higher proportion of males reported using a GABA agonist (26% versus 8%, χ^2^ = 7.95, *p* < 0.01).

**Table 2:** Treatments tried and recommended for juvenile primary fibromyalgia syndrome patients

Treatments	% of sample
Pharmacological treatments previously tried	
Daily non-steroidal anti-inflammatory drugs	27%
Selective serotonin reuptake inhibitors	17%
Tri-cyclic antidepressants	17%
Gamma-aminobutyric acid analogues	11%
Selective norepinephrine reuptake inhibitors	5%
Opioids	4%
Non-pharmacological treatments previously tried	
Physical therapy	21%
Herbals and supplements	9%
Therapeutic massage	16%
Mindfulness/meditation	12%
Chiropractic	6%
Acupuncture/acupressure	5%
Yoga	4%
Hypnosis	2%
Craniosacral therapy	1%
Treatments recommended or provided at baseline visit	
Psychoeducation on chronic pain	92%
Graduated aerobic exercise program	76%
Sleep hygiene education	70%
Physical therapy referral	57%
General counseling referral	53%
Medications	51%
Referral to pain clinic	46%
Cognitive-behavioral therapy referral	42%
Biofeedback referral	8%
Referral for psychiatric evaluation	4%
Referral for integrative medicine evaluation	3%
Referral to rehabilitation clinic	1%

About two-thirds (67%) of patients reported no prior use of any of the listed non-pharmacologic options. The most common non-pharmacologic treatment tried was PT (21%), and the least common was craniosacral therapy (two patients). Increasing age was modestly but reliably associated with reported use of some of the non-pharmacologic strategies, including meditation (*r*_pb_ = .20, *p* < .01), hypnosis (*r*_pb_ = .17, *p* = .03), and yoga (*r*_pb_ = .19, *p* = .01). The only significant sex difference observed was that males proportionately more often reported use of herbal remedies than females (22% versus 7%, χ^2^ = 6.06, *p* = .01).

### Initial pain and functional status

Pain scores at the baseline visit on average were in the moderate to severe range (mean = 6.3/10, SD = 2.4). Pain ratings did not significantly differ by sex or age. Scores on the functional disability and well-being measures were broad in range but on average indicated mild to moderate impairment (mean for CHAQ = .77, SD = .57; mean for subjective well-being rating = 5.1, SD = 2.2; mean for global rating of HRQOL = 3.0, SD = .84; median for current ACR class = Class 2). There were no age differences on the functioning and well-being measures. Males were found to be reliably more disabled than females based on the CHAQ measure (1.02 versus .70, *t* (199) = 4.12, *p* < .01). Males were also found to have worse HRQOL when compared to females (3.3 versus 2.9, *t* (194) = 2.01, *p* = .04).

### Treatments recommended

Table 2 also lists treatments recommended by rheumatologists at the baseline visit. In almost all cases (92%), providers reported doing education about chronic pain. Education on sleep hygiene and recommendations to begin a graduated aerobic exercise program also were common (70 and 76% of patients, respectively). For about half of patients (51%), medications were recommended. The most common referrals made included a referral to physical therapy (57% of patients), to general counseling (53% of patients), and to a pediatric or general pain clinic (46% of patients); patients were rarely referred to psychiatry (4%), integrative medicine (3%), or rehabilitation (1%). Only a minority of patients referred for outside services returned for follow-up with the rheumatologist during the study time period (36% of pain clinic referrals, 36% of counseling referrals, 26% of PT referrals, and 25% of CBT referrals).

### Treatments pursued

Of the 66 patients returning for follow-up visits during the study period, the majority (68%) of patients/parents reported doing most or all of the treatments recommended from the baseline visit; 28% reported doing some of the recommended treatments, and 4% reported doing none of the recommended treatments. The most common reasons for not pursuing the recommended treatment(s) included disinterest (16%), insurance failing to cover the treatment (8%), and the treatment not being available in the patient’s area (2%).

### Changes in symptoms and functional status over time

There was a reduced likelihood of reporting widespread pain over time since the baseline visit (b = − 0.57 ± 0.22, t (91) = − 2.53, *p* = 0.01). Otherwise, symptoms reported at the baseline visit remained relatively constant over time. Indicators of function and well-being were found to either worsen over time since the baseline visit (for CHAQ: *b* = 10 ± 0.05, t (91) = 2.05, *p* = 0.04; for subjective well-being: *b* = 0.65 ± 0.25, *t* (91) = 2.61, *p* = .01) or remain relatively consistent (for HRQOL: *b* = .05 ± 0.09, *t (*91) = 0.53, *p* = .59). Duration of symptoms prior to the first rheumatologist visit did not significantly moderate these results. Whether or not patients reported following initial treatment recommendations also did not moderate these findings. Worsening of subjective well-being over time was found to be even more pronounced in male patients (*b* = .1.53 ± 0.58, *t (*90) = 2.63, *p* = ..01. There were no other significant age or sex differences in changes in symptoms and functioning over time.

## Discussion

We sought to determine the demographic, clinical, and treatment characteristics of a representative sample of youth with JPFS patients who seek evaluation and treatment at pediatric rheumatology centers in North America. By virtue of using patient data from a registry in which multiple clinical sites participated, results of this study are thought to be relatively representative and generalizable to clinical samples of JPFS patients.

The majority of the JPFS patients in the registry cohort were White, non-Hispanic, adolescent females. These characteristics are similar to those observed in all studies of youth with juvenile fibromyalgia to date [2, 3, 5, 8, 19]. This may reflect demographic variation in family preferences and responses to symptoms of JPFS. Specifically, certain subgroups of patients with JPFS symptoms (e.g., ethnic minorities) may be less likely to seek treatment, or they may seek treatments in places not sampled by current studies. The demographic characteristics of this study population as well as other reported JPFS populations alternatively may suggest predisposing biological and/or environmental factors that are unique to this group [2]. Despite the typical disproportionate representation of females in the study sample, however, our male subsample was up to fivefold larger than that of other studies of youth with JPFS. Thus, this study allowed for some unique provisional comparisons by sex in the characteristics and treatment of JPFS.

Results from the current study indicate that pain in youth with JPFS is generally rated as moderate to severe in intensity. Stress, physical activity, and weather were identified by the majority of patients as exacerbating pain. Other symptoms found to be most frequently reported included fatigue, disordered sleep, and headaches. Conversely, symptoms more rarely endorsed (reported by less than one-third of the sample) included hypermobility, subjective soft tissue swelling, and IBS. Symptoms were found to be associated with moderate and enduring levels of impairment in functioning and well-being. The phenotypic characteristics observed in this study are largely consistent with data from other studies. In particular, pain scores from the current study (M = 6.3 ± 2.4/10) were comparable to, or slightly higher than, those reported in other studies of JPFS [8, 19]. Generalized aches, headaches and sleep disturbance were also found to be the most commonly reported symptoms (> 70%) in another cohort study [20], although relatively few patients in that study reported fatigue (20%, versus 84% in the current study). Most research on youth with JPFS has similarly found enduring moderate to severe functional impairment [19]. Results of our study therefore corroborate previous studies in their description of common presenting characteristics of patients with JPFS. Few sex differences were observed in these common presenting symptoms. However, males with JPFS reported higher levels of impairment in functioning and well-being. To our knowledge this has not been previously reported. This finding may have implications for the importance of recognition and diagnosis of JPFS symptoms in males presenting to pediatric rheumatology clinics.

Patients with JPFS reported about an 18 month time elapse on average between onset of symptoms and initial rheumatology evaluation. Other studies similarly have reported that symptoms may be present for many months prior to a correct diagnosis of JPFS [20, 21]. However, results of the current study were based on a multinational sample and 23 clinical sites. Thus, the observed delay in being evaluated in rheumatology is unlikely to be solely a reflection of barriers or trends that are peculiar to a particular site or region. During the waiting period, patients may see multiple providers and receive many tests or potentially risky or contraindicated treatment recommendations before receiving a diagnosis of JFPS. Data on prior treatments from the current study did suggest that a minority of patients had tried treatments with unknown or questionable efficacy (e.g., daily NSAIDs, opioids, chiropractic manipulation), whereas the majority had not yet been treated at all. Pain and functional limitations recently have been found to increase with a longer delay between symptom onset and appropriate diagnosis [22]. These findings collectively suggest that early identification and diagnosis is a critical unmet need for JPFS, one likely requiring optimized classification criteria and education of those providers most likely to first encounter these patients.

Treatments recommended or implemented in pediatric rheumatology for patients with JPFS were partly consistent with evidence-based care [23]. We are unable to determine from this study, however, the quality of the recommendations, education, and referrals provided. The most commonly prescribed or implemented interventions by pediatric rheumatologists based on this study included education on chronic pain, graduated aerobic exercise, sleep hygiene education, PT, and counseling. Aerobic and strength training exercises have evidence of being a safe and effective treatment for fibromyalgia symptoms [9]. Although the efficacy of general counseling for youth with fibromyalgia is unstudied, cognitive-behavioral therapy (CBT) has an established evidence base [8]. In the case of PT and CBT, however, motivation to participate may be low and result in poor follow-through and adherence, which in turn may impede symptom improvement. In the case of CBT for pain management, there may also be limited access to qualified providers to implement this treatment, and it is not yet known if remote options (e.g., telehealth, mobile applications) are equally efficacious replacements [24]. The majority of patients in the current study who returned for follow-up visits self-reported that they had followed most or all treatment recommendations, albeit we could not confirm the accuracy of these reports. Those reporting they did not follow recommendations, however, indicated motivation/interest as the primary barrier rather than access problems.

For about half of patients, pediatric rheumatologists also reported starting or changing medications. Although some medications (duloxetine, milnacipran, pregablin) are Federal Drug Administration (FDA) approved for treating fibromyalgia in adults, there are no FDA-approved medications for the treatment of JPFS. There also is weak agreement [25] on the benefits of medications prescribed off-label for fibromyalgia symptoms [7, 23]. Taken together, our study results suggest that pediatric rheumatology providers may benefit from collaborative efforts that help them recognize and treat JPFS using evidence-based, easily accessible, and practical treatment strategies. Historically such education has been minimally available during medical training [26]. Pediatric rheumatology fellowships should also consider increasing their focus on training in pharmacologic and non-pharmacologic pain management.

Results of this study should be interpreted with consideration of known limitations. Patients enrolled in the registry were seeking treatment in pediatric rheumatology, were able to speak and read English, and were enrolled by a subset of the total sites involved in the overall CARRA Legacy Registry. Thus, the resulting sample still may not be entirely reflective of the general population of youth with JPFS. Additionally, only about one-third of the baseline sample returned to contribute data at follow-up visits. The patients who returned for follow-up visits may be those whose symptoms are most refractory to treatment. If this were the case, the observed maintenance or worsening of symptoms and poor functioning over time may not accurately reflect trajectories of most patients.

Measures used to evaluate pain, functioning and well-being in the CARRA Legacy Registry also were selected for use across conditions seen in pediatric rheumatology. Although beneficial for cross-condition comparisons, some measures have less known specificity and utility for chronic pain. For example, whereas the Childhood Health Assessment Questionnaire is sensitive to the self-care and fine motor difficulties that can be common in other pediatric rheumatic diseases, it may not fully capture the range of physical, psychosocial, and academic impairments for youth with JPFS. Relatedly, a single measure of pain (pain intensity) was pragmatic to implement to quantify pain across conditions but does not fully reflect the features and dynamics of pain in JPFS. Use of other patient-reported outcome measures more commonly used in pediatric chronic pain studies to quantify pain and functional limitations, such as pain diaries and the Functional Disability Inventory, [27] may have produced different and more comprehensive findings.

## Conclusions

Overall, the current study further establishes that the main symptoms of JPFS are persistent and moderately disabling for many youth, with a subset of patients having minimal improvement despite at least some components of evidence-based care being implemented in pediatric rheumatology. Future research suggested by the study results should further explore gender differences among patients with JFPS, identification of barriers to timely diagnosis and treatment, and identification of effective treatment options to implement in the practice of pediatric rheumatology.

## Data Availability

The data that support the findings of this study are available from the Childhood Arthritis and Rheumatology Research Alliance (CARRA, Inc.), but restrictions apply to the availability of these data and so are not publicly available. Data are available upon reasonable request from and with permission of CARRA, Inc. Policies pertaining to data requests and sharing for CARRA, Inc. are available here at the following link: https://carragroup.org/UserFiles/file/CARRA-DATA-SAMPLE-SHARING-POLICY-04November2016.pdf

## Abbreviations

ACR: American College of Rheumatology
BMI: Body mass index
CARRA: Childhood Arthritis and Rheumatology Research Alliance
CBT: Cognitive behavioral therapy
CHAQ: Childhood Health Assessment Questionnaire
CRF: Case report form
FDA: Federal Drug Administration
GABA: Gamma-aminobutyric acid
HRQOL: Health-related quality of life
JIA: Juvenile idiopathic arthritis
JPFS: Juvenile primary fibromyalgia syndrome
MGA: Physician global assessment of disease activity
NRS: Numeric rating scale
NSAIDs: Nonsteroidal anti-inflammatory drugs
PT: Physical therapy
SNRIs: Serotonin norepinephrine reuptake inhibitors
SSRIs: Selective serotonin reuptake inhibitors

## References

[1] Buskila D, Press J, Gedalia A, Klein M, Neumann L, Boehm R (1993). Assessment of nonarticular tenderness and prevalence of fibromyalgia in children. J Rheumatol.

[2] Kashihar-Zuck S, Ting TV (2014). Juvenile fibromyalgia: current status of research and future developments. Nat Rev Rheumatol.

[3] Kashikar-Zuck S, King C, Ting TV, Arnold LM (2016). Juvenile fibromyalgia: different form the adult chronic pain syndrome?. Curr Rheumatol Rep.

[4] Weiss JE, Stinson JN (2018). Pediatric pain syndromes and noninflammatory musculoskeletal pain. Pediatr Clin N Am.

[5] Yunus MB, Masi AT (1985). Juvenile primary fibromyalgia syndrome. A clinical study of thirty-three patients and matched normal controls. Arthritis Rheum.

[6] Ting TV, Barnett K, Lynch-Jordan A, Whitacre C, Henrickson M, Kashikar-Zuck S (2016). 2010 American College of Rheumatology adult fibromyalgia criteria for use in an adolescent female population with juvenile fibromyalgia. J Pediatr.

[7] Gmuca S, Sherry DD (2017). Fibromyalgia: treating pain in the juvenile patient. Paediatr Drugs.

[8] Kashikar-Zuck S, Ting TV, Arnold LM, Bean J, Powers SW, Graham TB, Passo MH, Schikler KN, Hashkes PJ, Spalding S (2012). Cognitive behavioral therapy for the treatment of juvenile fibromyalgia: a multisite, single-blind, randomized, controlled clinical trial. Arthritis Rheum.

[9] Black WR, Kashikar-Zuck S (2017). Exercise interventions for juvenile fibromyalgia: current state and recent advancements. Pain Manag.

[10] Wolf F, Clauw DJ, Fitzcharles MA (2010). The American College of Rheumatology preliminary diagnostic criteria for fibromyalgia and measurement of symptom severity. Arthritis Care Res.

[11] Natter MD, Quan J, Ortiz DM (2013). An i2b2-based, generalizable, open source, self-scaling chronic disease registry. J Am Med Inform Assoc.

[12] Beighton P, Solomona L, Soskolone CL (1973). Articular hypermobility in an African population. Ann Rheum Dis.

[13] Smits-Engelsman B, Klerks M, Kirby A (2011). Beighton score: a valid measure for generalized hypermobility in children. J Pediatr.

[14] von Baeyer CL, Spagrud LJ, McCormick JC, Choo E, Neville K, Connelly MA (2009). Three new datasets supporting use of the numerical rating scale (NRS-11) for children's self-reports of pain intensity. Pain.

[15] Singh G, Athreya BH, Fries JF, Goldsmith DP (1994). Measurement of health status in children with juvenile rheumatoid arthritis. Arthritis Rheum.

[16] Nikiphorou E, Radner H, Chatzidionysiou K (2016). Patient global assessment in measuring disease activity in rheumatoid arthritis: a review of the literature. Arthritis Res Ther.

[17] Hochberg MC, Chang RW, Dwosh I (1992). The American College of Rheumatology 1991 revised criteria for the classification of global functional status in rheumatoid arthritis. Arthritis Rheum.

[18] Raudenbush SW, Bryk AS (2002). Hierarchical linear models: applications and data analysis methods.

[19] Kashikar-Zuck S, Cunningham N, Sil S (2014). Long-term outcomes of adolescents with juvenile-onset fibromyalgia in early adulthood. Pediatrics.

[20] Gedalia A, García CO, Molina JF, Bradford NJ, Espinoza LR (2000). Fibromyalgia syndrome: experience in a pediatric rheumatology clinic. Clin Exp Rheumatol.

[21] Cucchiaro G, Schwartz J, Hutchason A, Ornelas B (2017). Chronic pain in children: a look at the referral process to a pediatric pain clinic. Int J Pediatr.

[22] Palermo TM, Slack M, Zhou C, Aaron R, Fisher E, Rodriguez S (2018). Waiting for a pediatric chronic pain clinic evaluation: a prospective study characterizing waiting times and symptom trajectories. J Pain.

[23] Macfarlane GJ, Kronisch C, Dean LE (2017). EULAR revised recommendations for the management of fibromyalgia. Ann Rheum Dis.

[24] Fisher E, Law E, Palermo TM, Eccleston C. Psychological therapies (remotely delivered) for the management of chronic and recurrent pain in children and adolescents. Cochrane Database Syst Rev. 2015;3(3):CD011118. 10.1002/14651858.CD011118.pub2 PMC4833498 25803793

[25] Andrews J, Guyatt G, Oxman AD (2013). GRADE guidelines: 14. Going from evidence to recommendations: the significance and presentation of recommendations. J Clin Epidemiol.

[26] Mezei L, Murinson BB (2011). Pain education in north American medical schools. J Pain.

[27] Flowers SR, Kashikar-Zuck S (2011). Measures of juvenile fibromyalgia: functional disability inventory (FDI), modified fibromyalgia impact questionnaire-child version (MFIQ-C), and pediatric quality of life inventory (PedsQL) 3.0 rheumatology module pain and hurt scale. Arthritis Care Res (Hoboken).

