# Correction to: Demographic, clinical, and treatment characteristics of the juvenile primary fibromyalgia syndrome cohort enrolled in the Childhood Arthritis and Rheumatology Research Alliance Legacy Registry

**DOI:** 10.1186/s12969-019-0395-5

**Published:** 2020-01-21

**Authors:** Jennifer E. Weiss, Kenneth N. Schikler, Alexis D. Boneparth, Mark Connelly, and For the CARRA Registry Investigators

**Affiliations:** 1 Hackensack University Medical Center, 30 Prospect Ave, Hackensack, NJ 07601 USA; 2 University of Louisville School of Medicine, Louisville, KY 40292 USA; 3 New York-Presbyterian Medical Center, New York, NY 10032 USA; 4 Children’s Mercy Kansas City, 2401 Gillham Road, Kansas City, MO 64108 USA

**Correction to: Pediatr Rheumatol**

**https://doi.org/10.1186/s12969-019-0356-z**

Following publication of the original article [1], we have been notified that the corresponding author’s given name is spelled incorrectly. The given name, thus, should be as follows:

Jennifer E. Weiss
